# Population Specific Impact of Genetic Variants in *KCNJ11* Gene to Type 2 Diabetes: A Case-Control and Meta-Analysis Study

**DOI:** 10.1371/journal.pone.0107021

**Published:** 2014-09-23

**Authors:** Nagaraja M. Phani, Vasudeva Guddattu, Ravishankara Bellampalli, Venu Seenappa, Prabha Adhikari, Shivashankara K. Nagri, Sydney C. D′Souza, Gopinath P. Mundyat, Kapaettu Satyamoorthy, Padmalatha S. Rai

**Affiliations:** 1 Division of Biotechnology, School of Life Sciences, Manipal University, Manipal, Karnataka, India; 2 Department of Statistics, Manipal University, Manipal, Karnataka, India; 3 Department of Medicine, Kasturba Medical College, Manipal University, Mangalore, Karnataka, India; 4 Department of Medicine, Kasturba Medical College, Manipal University, Manipal, Karnataka, India; CAEBi, Spain

## Abstract

**Background and Objectives:**

Potassium inwardly rectifying channel, subfamily J, member 11 (*KCNJ11*) gene have a key role in insulin secretion and is of substantial interest as a candidate gene for type 2 diabetes (T2D). The current work was performed to delineate the genetic influence of *KCNJ11* polymorphisms on risk of T2D in South Indian population through case-control association study along with systematic review and meta-analysis.

**Methods:**

A case-control study of 400 T2D cases and controls of South Indian origin were performed to analyze the association of *KCNJ11* polymorphisms (rs5219, rs5215, rs41282930, rs1800467) and copy number variations (CNV) on the risk of T2D. In addition a systematic review and meta-analysis for *KCNJ11* rs5219 was conducted in 3,831 cases and 3,543 controls from 5 published reports from South-Asian population by searching various databases. Odds ratio with 95% confidence interval (CI) was used to assess the association strength. Cochran's Q, I^2^ statistics were used to study heterogeneity between the eligible studies.

**Results:**

*KCNJ11* rs5215, C-G-C-C haplotype and two loci analysis (rs5219 vs rs1800467) showed a significant association with T2D but CNV analysis did not show significant variation between T2D cases and control subjects. Lower age of disease onset (P = 0.04) and higher body mass index (BMI) (P = 0.04) were associated with rs5219 TT genotype in T2D patients. The meta-analysis of *KCNJ11* rs5219 on South Asian population showed no association on susceptibility to T2D with an overall pooled OR = 0.98, 95% CI = 0.83–1.16. Stratification analysis showed East Asian population and global population were associated with T2D when compared to South Asians.

**Conclusion:**

*KCNJ11* rs5219 is not independently associated with T2D in South-Indian population and our meta-analysis suggests that *KCNJ11* polymorphism (rs5219) is associated with risk of T2D in East Asian population and global population but this outcome could not be replicated in South Asian sub groups.

## Introduction

Insulin secretion induced by glucose comprises of an intricate network of regulatory mechanisms involving glucose metabolism by pancreatic β-cell and electrical activity controlled by ion channels of plasma membrane. A key role of ATP-sensitive potassium (KATP) channel is to control insulin secretion by glucose and to pair metabolism of glucose with electrical activity of the membrane [1]. KATP channel is made up of two structurally unrelated subunits of octameric protein complexes with four pores forming inwardly-rectifying K^+^ channel. Potassium inwardly rectifying channel, subfamily J, member11 (*KCNJ11*) subunits linked to four high affinity sulfonylurea receptor (SUR1, *ABCC8*) subunit [2]. *KCNJ11* has attracted considerable interest in recent years as candidate gene for Type 2 diabetes (T2D) and has a reported 180 single nucleotide polymorphisms (SNPs) in NCBI (National Centre for Biotechnology Information) database for humans. *KCNJ11* polymorphisms rs5219 (E23K), rs1800467 (L270V), rs5215 (V337I), rs41282930 (S385C) are four common missense polymorphisms that have been observed in *KCNJ11* gene and has shown to influence the risk of T2D in multiple studies including recent large scale genetic studies [2]–[4], [7]. Initial reports of Hani et al., (1998) and Yamada et al., (2001) were inconsistent with *KCNJ11* rs5219 (C/T) variant association with T2D [5], [6]. Gloyn et al., (2003) and Love et al., (2003) confirmed the association of *KCNJ11* rs5219 (C/T) polymorphism and the susceptibility to T2D in Caucasian subjects [7], [8]. Reports from *in vitro* experiments have revealed that Kir6.2 with K23 type allele showed an increased threshold of ATP concentration for the release of insulin [9], [10]. Consistent with these reports, Nielsen et al., (2003) and Florez et al., (2004) observed a considerable decrease in insulin release during an oral glucose tolerance test (OGTT) with subjects carrying *KCNJ11* T23 allele [11], [12]. The role of *KCNJ11* polymorphism (rs5219) with T2D is less explored in non-Caucasian populations. Reports from earlier studies have shown that *KCNJ11* polymorphism (rs5219) appears fairly common in Asian subjects and to a lesser degree in individuals of African descent. In this population, small and medium size studies were performed and contradictory results were reported for the association of this variant with T2D [6], [12]–[16]. The limitations of the published studies with respect to sample number and ethnic diversity restricts exploration of the effect of rs5219 on susceptibility to T2Dand limited number of studies may be insufficient to arrive at a discernible conclusion. Adequate comprehensive meta-analyses and genome wide association studies (GWAS) are yet to be performed to decisively determine the association of *KCNJ11* polymorphism (rs5219) and T2D in South Asian populations, as has been performed in East Asian and Caucasian populations. Global meta-analyses undertaken so far on *KCNJ11* polymorphism (rs5219) have collected all the studies and this may not be suitable for Asians and Caucasians as they may differ in their genetic predisposition to T2D [17]. Asian population differs in their ethnic background; for example, the South Asian Indians are primarily of Indo-European and Dravidian ancestries who are quite different from East Asian populations. Wang et al., (2009) has reported influence of differences in ethnicity on predisposition to human diseases [18]. In the last few years, there has been an increase in the number of studies on Asian population and there is a need to collectively analyze this data. In this study, we have performed a case-control association analysis for *KCNJ11* polymorphisms and CNV for T2D risk in a large South Indian population. A systematic review and meta-analysis of *KCNJ11* rs5219 gene polymorphism on all available studies from South Asian populations was also performed and we compared our results with the meta-analysis of East Asian population and global population to explore the possibilities of ethnic differences in relation to *KCNJ11* polymorphism (rs5219) and T2D risk.

## Materials and Methods

### Case-control study

#### Ethics statement

The study was approved by the institutional review board, Kasturba Hospital ethical committee of Manipal University and we obtained written informed consent from all the study participants.

#### Study population

The study involved a total of 400 unrelated individuals as cases and 400 controls of South Indian origin recruited from the Kasturba Hospital, Manipal and Mangalore, Karnataka state, India. The cases and control subjects were recruited on the basis of our inclusion criteria. For cases (a) clinically diagnosed T2D patients as defined by world health organization (WHO) criteria (b) disease onset age>30 years (c) belonging to South Indian origin. For controls (a) fasting glucose <126 mg/dl and glycated hemoglobin (HbA1c) <6.0% (b) no history of diabetes in the first and second degree relatives (c) age>40 years (d) belonging to South Indian origin.

#### Anthropometric measurements and metabolic assays

The general anthropometric parameters considered for the study includes height (m), weight (kg), which were done as per standardized protocols, and BMI was calculated. Biochemical measurements including levels of glycated hemoglobin (HbA1c), fasting plasma glucose (FPG), total cholesterol (TC), Low density lipoprotein (LDL) cholesterol, High density lipoprotein (HDL) cholesterol, and triglycerides (TG) were determined by following manufacturer's instructions using Hitachi 912 auto-analyzer (Roche, Basel, Switzerland). HbA1c levels were estimated in whole blood using Cobas integra 512 clinical chemistry auto-analyzer following manufacturer's instructions.

#### Genotyping of *KCNJ11* polymorphisms

The Genomic DNA was extracted from peripheral blood by conventional phenol-chloroform method [19]. Genotyping of *KCNJ11* polymorphisms (rs5219, rs5215, rs41282930, rs1800467) was carried out using tetra primer amplification refractory mutation system (TETRA-ARMS). This method uses two primer sets in which the outer primers are specific to amplify a larger fragment of DNA containing the SNP and the inner allele specific primers are specific to amplify two different alleles in a single polymerase chain reaction (PCR). The differing sizes of amplicon representing the presence of alleles were resolved on agarose gel electrophoresis. Online primer design program by Ye et al., (2001) (http://cedar.genetics.soton.ac.uk) was used to design the primers [20]. The primers were designed by limiting the fragment sizes to the range of 150–400 bp and the ratio of the allelic bands to 1.2–1.5. PCR was performed in a total volume of 10µl containing 30 ng of template DNA, 10 pmol of each inner primer, 1 pmol of each outer primer, 200µM dNTPs, 1X PCR buffer (1.5 mM MgCl_2_;10 mM 50 mMKCl; Tris HCl, pH 8.8; 0.1% Triton X-100), and 1 U of *Taq* polymerase (Finnzymes, USA). PCR condition includes initial denaturation of 95°C for 5 minutes, 35 cycles of 95°C for 30 seconds, 64°C for 45 seconds and 72°C for 45 seconds and a final extension of 72°C for 10 minutes. Primer sequences and amplicon size are given in Table S1

#### Quantitative real time PCR for CNV analysis

Quantitative real time PCR (qPCR) was used to detect CNVs in *KCNJ11* gene of T2D and control subjects. 2^−ΔΔCt^ method was used for relative quantification of CNVs [21]. The difference in threshold cycles (Ct) between the test gene and RNaseP (Control gene) was determined from two experiments, each performed in duplicate. The copy number variation in the target gene was determined relative to the level of RNaseP. Standard curves for the primers of the target region and control regions were generated prior to the copy number assay to determine their PCR efficiency. qPCR was carried out using SYBR Green Master mix from Invitrogen on 7500 Fast Real-Time PCR system following the manufacturer's instructions and cycling conditions.

#### Statistical analysis

Quanto version 1.2.4 (Gauderman et al., 2009) was used for sample size calculation using minor allele frequency data from dbSNP database (http://www.ncbi.nlm.nih.gov/projects/SNP/) [22]. The study achieved 80% power at P value 0.05 and Pearson's Goodness of fit chi-squared test was used to determine whether the polymorphism is in Hardy–Weinberg equilibrium (P>0.05) in controls before the analysis. Association analysis of the SNP with T2D for difference in allele and genotype frequency between diabetic and control subjects was done by using Pearson's chi-squared test. Logistic regression analysis was used to further test the association of SNP with T2D as measured by odds ratio (OR) and corresponding 95% confidence interval (CI) after adjusting for age, sex and BMI as covariates. Association analysis was also performed assuming co-dominant, dominant, and recessive models. In our analysis, the common homozygote genotype in the control population was considered as the reference category. Comparison of clinical variables between cases and controls were performed using Student's t- test and are represented as mean ± SD. The T2D subjects and controls were further analyzed for haplotype effects of four loci using UNPHASED (version 2.404) [23]. Analysis of covariance (ANOCVA) was used to test for the significant interaction of quantitative traits with genotype using univariate general linear regression model to adjust for age, sex and BMI as covariates. All statistical analysis was performed using SPSS version 16 for windows (SPSS, Chicago, Illinois, USA) and SNPstat Software (Sole et al., 2006) [24]. A P value of<0.05 (two tailed) was considered statistically significant.

### Systematic review and Meta-analysis of *KCNJ11* rs5219 gene polymorphism

#### Search strategy and selection criteria

A broad search was performed for reports on *KCNJ11*polymorphism (rs5219) and T2D in PubMed, Scopus, Science direct and Embase for all relevant articles pertaining to South Asian and East Asian population. The keywords for searching included Kir6.2/potassium inwardly rectifying channel, subfamily J, member 11/*KCNJ11* rs5219/in combination with*KCNJ11* and T2D and T2D in South Asia/India/Pakistan. We searched for all possible combination of keywords one at a time. All articles published till November 2013 was included. To further identify eligible studies, reference lists from the retrieved articles were examined. Potentially relevant articles from PubMed were extracted using “Related Articles” option.

#### Data extraction from eligible studies

Published manuscripts which met the following criteria were selected: 1) study participants must be from South Asian countries, 2) study should be published in peer reviewed journals with original data, 3) study should investigate the association of *KCNJ11* polymorphism (rs5219) with T2D, 4) study design should conform to cases vs controls, 5) study should provide allelic frequencies, genotypic frequencies and their distribution in cases and controls and odds ratio (OR) with 95% CI, 6) study should use World Health Organization Criteria (WHO) or American Diabetes Association criteria (ADA) for diagnosis of T2D patients, 7) non-diabetic individuals as controls should be included and 8) method of genotyping used should be explained or linked to a reference. We have excluded studies for 1) overlapping and insufficient data and 2) family based and case only studies or review articles. Guidelines from meta-analysis of observation studies in epidemiology (MOOSE) group was followed to extract the following information: name of first author, year of publication, ethnic background of study subjects, work plan, criteria followed for diagnosis of T2D, source of control, mean age of case and controls, method of genotyping, single nucleotide polymorphisms studied, P value for Hardy Weinberg Equilibrium test (HWE), allelic and genotypic frequency and their distribution in cases and controls, adjusted odds ratio (OR)and 95% CI. Supplementary materials if available were scanned for data extraction. The studies were reviewed by two independent reviewers and disagreements concerning inclusion/exclusion of studies or risk estimates were resolved by consensus. Cohen's kappa coefficient was used to assess the agreement between the reviewers for inclusion/exclusion of studies [25].

#### Study quality assessment

Newcastle-Ottawa scale (NOS) was used to assess methodological quality of the included nonrandomized studies. Three major parameters were taken into consideration to evaluate the quality: 1) selection of groups of the studies 2) comparability 3) assessment of the outcome or exposure. The maximum score could be 9 points for each study representing the highest methodological quality [26].

#### Statistical analysis

We used OR as the measure of association between *KCNJ11* polymorphism (rs5219) and T2D with its corresponding 95% CI. For every allele OR of the risk allele (i.e. C allele vs T allele) was compared between cases and controls. To assess for heterogeneity within and between the eligible studies, Cochran's Q-statistic which followed a chi-square distribution with *k*−1 degrees of freedom (df) was utilized. P<0.05 was regarded as statistically significant [27]. I^2^ statistic was reported as a measure of heterogeneity in odds ratio between the studies included in the meta-analysis. An I^2^ value of <25% indicates low heterogeneity, 25–50%moderate heterogeneity, 50–75% high heterogeneity and>75% extreme heterogeneity [28]. Pooled OR and 95% CI was estimated using random effects model which can look out both between study and within study variability (Der Simonian and Laird) [29]. Tau Square (τ^2^) was reported as a measure of between study variance. The smaller the value of τ^2^, the smaller is the variance. Sensitivity analysis was performed to evaluate the weight of each individual study on the pooled OR. All analyses were performed with MetaXL1.3 tool for meta-analysis in Microsoft excel.

## Results

### Case-control study

#### Characteristics of study subjects

A total of 400 T2D subjects and 400 control subjects of South Indian origin were enrolled for this study. Table 1 summarizes the clinical and physical characteristics of all the subjects included in the study. T2D subjects had significantly higher FPG, HbA1c, TC and TG while they exhibited a lower HDL level when compared with the control subjects.

**Table 1:** Clinical characteristics of study population.

Characteristics	T2D Subjects (n = 400)	Control Subjects (n = 400)	*P*
Age (years)	55.24±11.2	53.9±14.2	0.125
Age at diagnosis (years)	49.2±10.3	-	
BMI (kg/m^2^)	24.8±3.6	24.9±3.2	0.408
HbA1c (%)	8.62±1.9	5.2±1.4	<0.05*
FPG (mg/dl)	160.0±55.8	89.4±13.7	<0.05*
TC (mg/dl)	179.1±36.9	168.4±30.1	<0.05*
TG(mg/dl)	143.6±48.9	102.9±25.7	<0.05*
HDL cholesterol (mg/dl)	30.7±11.25	46.6±8.2	<0.05*
LDL cholesterol (mg/dl)	121.9±36.0	108.2±57.1	0.95

Data for quantitative variables are mean ± SD, *P value <0.05 is considered significant.

Abbreviations: BMI-Body mass index, HbA1c-Glycated hemoglobin, FPG-Fasting plasma glucose, TC-Total cholesterol, TG-Triglycerides, HDL-High density lipoproteins, LDL-Low density lipoproteins.

#### Association analysis

Out of four *KCNJ11* variants (rs5219, rs5215, rs41282930, rs1800467) only rs5215 variant showed statistically significant association with T2D susceptibility. We observed a considerable difference in the distribution pattern of TT genotype of rs5215 between T2D subjects and controls (22.2% vs 11.2%) and this SNP showed a significant increased risk with T2D (OR = 1.33, 95% CI = 1.0–1.6, P = 0.005) under an co-dominant model and we observed a synergistic protective effect in recessive model with an OR = 0.42, 95% CI = 0.28–0.62, P = 0.0001 (Table 2).

**Table 2:** Genotypic, allelic distribution and association analysis of *KCNJ11* rs5219, rs5215, rs41282930, rs1800467gene polymorphisms and risk of T2D under different genetic models.

Gene/SNP	Genotype/Allele	Case (%) n = 400	Control (%) n = 400	Co-Dominant CC vs CT, TT	Dominant CT+TT vs CC	Recessive TT vs CC+CT
*KCNJ11* rs5219	CC	184 (46.3)	176 (44)	1.19(0.6–2.1) *P*-0.59	1.06(0.6–1.7) *P*-0.84	0.75(0.39–1.47) *P*-0.41
	CT	113 (28.2)	168 (42)			
	TT	102 (25.5)	56 (14)			
	Allele C	0.6	0.65			
	Allele T	0.4	0.35			
*KCNJ11 rs5215*	CC	182(45.5)	185(46.2)	1.33(1.0–1.6) *P*-0.005*	0.97(0.73–1.28) *P*-0.83	0.42(0.28–0.62) *P*-0.0001*
	CT	125(31.2)	170(42.5)			
	TT	93(22.2)	45(11.2)			
	Allele C	0.61	0.68			
	Allele T	0.39	0.32			
*KCNJ11 rs41282930*	CC	359(89.8)	387(96.8)	0.32(0.1–0.59) *P*-0.0001*	0.29(0.16–0.56) *P*-0.0001*	1.0(0.06–16.04) *P*-0.9
	CG	40(10)	12(3)			
	GG	1(0.2)	19(0.2)			
	Allele C	0.05	0.02			
	Allele G	0.95	0.98			
*KCNJ11 rs1800467*	CC	135(33.8)	155(38.8)	1.02(0.8–1.24) *P*-0.80	0.81(0.60–1.07) *P-*0.14	1.18(0.88–1.57) *P*-0.27
	CG	130(32.5)	95(23.80)			
	GG	135(33.5)	150(37.5)			
	Allele C	0.5	0.51			
	Allele G	0.5	0.49			

*P value <0.05 is considered significant, 95% CI that did not include unity is statistically significant.

*P* values were adjusted for age, gender and BMI.

#### Two-locus analysis

The above results of the present work prompted us to go for a two-locus analysis between the variants to look for any possible interactions among the variants on susceptibility to T2D. Two locus analysis between the variants rs5215 and rs5219, rs5219 and rs41282930, rs5215 and rs41282930, rs5215 and rs1800467, rs41282930 and rs1800467 did not show any statistically significant association with susceptibility with T2D (Data not shown). However two-locus analysis between rs5219 and rs1800467 showed a considerable collective effect on T2D. For this analysis the study subjects were grouped into two groups based on the genotypes of rs5219 variant: C/C subjects and C/T+T/T subjects. In the presence of C/C genotype the rs1800467 variant showed an increased susceptibility to T2D with an OR = 1.62, 95%CI = 1.03–2.53 P = 0.03. Where as in the presence of C/T+T/T genotype the rs1800467 variant showed an insignificant risk with an OR = 0.96, 95%CI = 0.65–1.4 P = 0.83 (Table 3).

**Table 3:** Two loci analysis involving the genotypes of the variants rs5219 and rs1800467 of *KCNJ11* gene with type2 diabetes.

Genotype of rs5219 vs rs1800467	T2D Subjects	Control Subjects	OR (95% CI)
C/C Subjects (rs5219)			
C/C	50(27%)	66(38%)	1.62(1.03–2.53)
(C/G+G/G)	134(73%)	109(62%)	*P* = 0.03
C/T+T/T Subjects (rs5219)			
C/C	83(39%)	89(40%)	0.96(0.65–1.4)
(C/G+G/G)	132(61%)	136(60%)	*P* = 0.83

#### Haplotype analysis

The association of haplotypes of four *KCNJ11* loci (rs5219, rs5215, rs41282930, rs1800467) was tested using UNPHASED which is a program that uses an algorithm to obtain a maximum Likelihood estimates of case and control haplotype frequency under an alternative and null hypothesis are summarized in table 4. Haplotype C-G-C-C was shown to be associated with T2D susceptibility with an OR = 1.46, 95% CI = 1.35–3.54 P = 0.05 (Table 4).

**Table 4:** Haplotype Analysis of the *KCNJ11 rs5219, rs5215, rs4128930, rs1800467* polymorphisms using a reference haplotypes.

Type	Haplotype	Case frequency (n = 400)	Control frequency (n = 400)	OR(95%CI)	χ^2^	*P*
Reference haplotype	C-A	0.21	0.20	1	0.23	0.63
H1	T-G	0.207	0.251	0.80(0.59–1.08)	4.01	0.045*
Reference haplotype	C-A-C	0	0.01	1	2.98	0.08
H2	T-G-G	0.236	0.198	3.69(0.01–1.37)	4.01	0.045*
Reference haplotype	C-A-C-C	0	0.004	1	0.62	0.429
H3	C-G-C-C	0.01	0	1.46(1.35–3.54)	3.83	0.050*

Haplotype frequencies were compared using Chi squared test. *P-value <0.05 is considered significant. Haplotypes within each haplogroups (H1, H2, and H3) which showed statistically significance are represented in the table.

#### Correlation between KCNJ11 genotype and T2D related quantitative traits

In order to explore for the possible mechanism of these variants on the susceptibility to T2D we tested the association of the *KCNJ11*polymorphisms (rs5219, rs5215, rs41282930, rs1800467) on T2D related quantitative traits (FPG, HbA1c, TC, TG, HDL, LDL, TC/HC, age at diagnosis and BMI) using ANOCVA with univariate general linear regression model to adjust for age, sex and BMI as covariates. *KCNJ11*rs5219 T/T genotype showed an increased BMI (30.8±33.1, P = 0.04) and an early age of disease onset (47.0±10.2, P = 0.04) in T2D subjects. *KCNJ11* rs1800467 G/G genotype showed an increased TC level (185.2±34.4, P = 0.01) in T2D subjects when compared to other two genotypes (Table 5, Table 6).

**Table 5:** Effects of *KCNJ11* rs5219, rs5215, rs41282930, rs1800467 gene polymorphisms on clinical and biochemical parameters in T2D patients.

Gene/SNP	CASES									
		Age (yrs)	Diagnosis age (yrs)	FPG mg/dl	HbA1c %	TC mg/dl	TG mg/dl	HDLmg/dl	LDL mg/dl	BMI (kg/m^2^)
*KCNJ11 rs5219*	CC	55.7(±11.8)	50.7(±10.8)	203.9(±113.3)	8.6 (±1.8)	181.2(±35.8)	145.5(±44.6)	30.0(±10.6)	122.3(±37.2)	25.9(±2.64)
	CT	56.6 (±10.6)	49.5 (±9.6)	218.1(±138.4)	8.3(±1.85)	181.8(±36.3)	140.3(±48.2)	32.2(±11.8)	123.3(±35.5)	25.5 (±2.3)
	TT	53.0 (±10.4)	47.0 (±10.2)	196.9(±44.4)	8.9 (±2.3)	172.7(±38.9)	144.7(±56.3)	30.0(±11.1)	116.3(±34.1)	30.8(±33.1)
		*P*-0.05	***P*** -0.04*	*P-*0.32	*P-*0.1	*P*-0.09	*P*-0.68	*P*-0.24	*P-*0.36	***P*** -0.04*
*KCNJ11* rs5215	CC	55.29(±11.6)	50.0(±10.1)	200.7(±148.8)	8.8(±2.1)	178.8(±36.8)	141.1(±48.1)	30.8(±10.9)	121.8(±33.0)	28.5(±2.7)
	CT	55.06(±10.8)	48.75(±10.9)	211.0(±130.1)	8.4(±1.9)	183.1(±35.4)	145.2(±46.6)	30.8(±11.0)	121.1(±37.6)	25.6(±2.4)
	TT	55.4(±11.3)	49.0(±9.65)	209.6(±148.8)	8.3(±1.6)	174.2(±38.8)	146.4(±53.5)	30.4(±12.2)	120.0(±39.6)	26.0(±2.6)
		*P-*0.97	*P*-0.65	*P*-0.67	*P-*0.06	*P*-0.21	*P*-0.63	*P*-0.96	*P*-0.92	*P-*0.26
*KCNJ11 rs41282930*	CC	60.5(±0.7)	57.5(±2.12)	238.5(±38.8)	7.8(±1.4)	219.5(±48.7)	133.5(±21.9)	27.0(±11.3)	139.0(±39.5)	21.1(±2.9)
	CG	52.8(±12.2)	48.5(±10.3)	195.5(±45.3)	8.6(±1.7)	184.2(±38.6)	149.0(±59.2)	30.6(±9.7)	130.7(±37.2)	26.7(±2.6)
	GG	55.4(±11.1)	49.2(±10.4)	207.0(±113.9)	8.6(±2.0)	178.3(±36.6)	143.1(±47.8)	30.7(±11.4)	120.0(±35.8)	27.1(±17.7)
		*P*-0.30	*P*-0.50	*P*-0.75	*P*-0.84	*P*-0.19	*P*-0.73	*P*-0.89	*P-*0.16	*P*-0.87
*KCNJ11* rs1800467	CC	55.2(±11.3)	49.6(±11.2)	207.1(±126.0)	8.5(±1.9)	179.7(±36.6)	144.0(±47.9)	30.6(±10.3)	122.6(±34.7)	25.7(±2.4)
	CG	54.5(±11.0)	47.7(±9.4)	209.3(±129.5)	8.6(±2.0)	172.1(±38.7)	143.7(±53.9)	29.9(±12.5)	115.0(±36.7)	27.9(±2.7)
	GG	55.8(±11.4)	50.2(±9.9)	201.8(±55.0)	8.7(±1.9)	185.2(±34.4)	143.2(±45.0)	31.6(±10.7)	125.6(±36.1)	27.4(±2.4)
		*P*-0.62	*P*-0.28	*P*-0.85	*P*-0.68	*P*-0.01*	*P*-0.9	*P*-0.45	*P*-0.05	*P-*0.50

Values are mean ± SD and were compared using ANOVA,*P<0.05 is considered statistically significant.

Abbreviations: FPG- Fasting plasma glucose, HbA1c-Glycated hemoglobin, TC-Total cholesterol, TG-Triglycerides, HDL-High density lipoproteins, LDL- Low density lipoproteins, BMI- Body mass index.

**Table 6:** Effects of *KCNJ11* rs5219, rs5215, rs41282930, rs1800467 gene polymorphisms on clinical and biochemical parameters in normal healthy controls.

Gene/SNP	CONTROLS								
		Age (yrs)	FPG mg/dl	HbA1c %	TC mg/dl	TG mg/dl	HDLmg/dl	LDL mg/dl	BMI (kg/m^2^)
*KCNJ11 rs5219*	CC	47.2 (±15.3)	89.3 (±12.9)	5.2 (±3.7)	166.2(±31.1)	105.4(±26.7)	46.2 (±7.5)	107.5(±51.8)	22.7 (±2.0)
	CT	47.0 (±14.7)	89.0 (±14.9)	5.3 (±3.8)	171.2(±29.8)	100.3(±24.9)	46.5 (±9.2)	110.2(±68.4)	22.9 (±1.9)
	TT	44.7 (±17.1)	90.8 (±12.4)	5.0 (±0.7)	167.2(±27.7)	102.9(±24.4)	48.5 (±6.7)	104.6(±29.1)	23.4 (±1.7)
		*P*-0.56	P-0.68	P-0.68	P-0.28	P-0.17	P-0.16	P-0.79	P-0.06
*KCNJ11* rs5215	CC	48.2(±14.2)	89.0(±13.6)	5.2(±3.6)	166.9(±31.5)	103.7(±25.7)	46.4(±7.0)	110.4(±65.5)	23.0(±1.9)
	CT	45.5(±16.6)	90.0(±14.5)	5.4(±3.8)	167.8(±28.5)	102.9(±25.6)	46.7(±9.7)	108.2(±52.7)	22.6(±2.0)
	TT	44.8(±14.0)	88.3(±10.8)	5.0(±0.7)	177(±29.4)	99.6(±26.5)	47.5(±6.7)	99.6(±28.9)	23.0(±1.7)
		P-0.14	P-0.68	P-0.77	P-0.12	P-0.63	P-0.69	P-0.52	P-0.15
*KCNJ11 rs41282930*	CC	45.0(±0.0)	86.0(±0.0)	5.7(±0.0)	187(±0.0)	76.0(±0.0)	51.0(±0.0)	119(±0.0)	22.1(±0.0)
	CG	40.5(±16.8)	94.1(±16.0)	4.9(±0.8)	177.2(±21.7)	93.0(±25.0)	48.5(±5.9)	110.1(±25.7)	21.7(±1.7)
	GG	47.0(±15.3)	89.2(±13.6)	5.2(±3.5)	168.8(30.3)	103.3(±25.7)	46.5(±8.3)	108.1(±57.8)	22.9(±1.9)
		P-0.35	P-0.46	P-0.9	P-0.48	P-0.23	P-0.62	P-0.97	P-0.10
*KCNJ11* rs1800467	CC	46.0(±15.6)	89.1(±14.1)	5.4(±3.9)	168.8(±30.8)	104.9(±25.5)	46.0(±6.9)	109.1(±53.3)	23.0(±2.1)
	CG	48.0(±15.2)	89.3(±13.8)	5.6(±5.0)	167.9(±27.8)	103.9(±25.9)	46.9(±7.1)	102.2(±27.7)	22.4(±1.8)
	GG	46.8(±15.1)	89.6(14.1)	4.9(±0.7)	168.5(±31.0)	100.2(±25.8)	47.1(±9.9)	111.2(±72.5)	23.0(±1.8)
		P-0.59	P-0.9	P-0.29	P-0.9	P-0.25	P-0.52	P-0.47	P-0.38

Values are mean ± SD and were compared using ANOVA,*P<0.05 is considered statistically significant.

Abbreviations: FPG- Fasting plasma glucose, HbA1c-Glycated hemoglobin, TC-Total cholesterol, TG-Triglycerides, HDL-High density lipoproteins, LDL- Low density lipoproteins, BMI- Body mass index.

#### Copy number variation (CNV) analysis

In the current study we also analyzed CNVs in the *KCNJ11* gene in 50 T2D and 50 control subjects. No deletions or duplications were observed in the control samples; however two T2D samples were found to be deleted.

### Systematic review and meta-analysis

#### Characteristics of eligible studies

**Figure 1:**
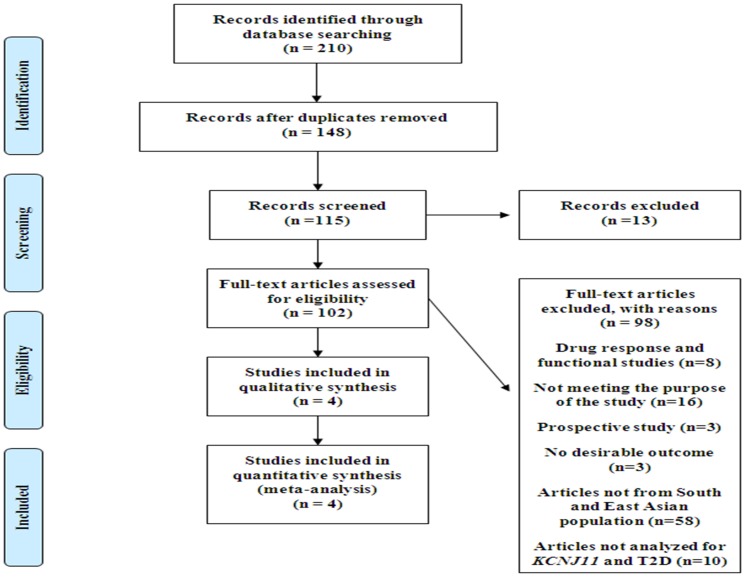
Flow diagram of study selection for *KCNJ11* polymorphism (rs5219).

A total of 471 articles were identified for *KCNJ11*polymorphism (rs5219) and T2D by searching 4 databases (PubMed, Scopus, Science direct and Embase). Relevant titles and summaries were identified and 115 were chosen from 210 articles after removing duplicates, reviews and letters. Of the 115 articles we only included case reports, drug response studies, functional studies of *KCNJ11* gene and prospective studies. 72 articles were retrieved for detailed evaluation out of which 58 articles were discarded as the study population was different and 10 articles did not analyze *KCNJ11* polymorphism (rs5219) with T2D. The remaining 4 articles in addition to the present study were included in the final qualitative analysis (Figure 1). The observed Cohen's kappa for the agreement between reviewers was 0.86. Quality of the data obtained for qualitative synthesis (meta-analysis) was guaranteed by evaluating the studies using NOS scale. Three out of four studies (Gupta et al., 2010, Chauhan et al., 2010, Sanghera et al., 2008) scored 7 points and one study (Rees et al., 2011) scored 6 points out of 9 indicating good study quality (Table S2). Table 7 depicts these distinct 5 studies included in the qualitative analysis [31]–[34]. All studies were in HWE. The 5 articles involved in the quantitative analysis contain a total of 3,831 cases of T2D and 3,543 controls.

**Table 7:** Characteristics of the studies examining the associations of *KCNJ11* gene polymorphism (rs5219) and T2D included in the meta-analysis (n = 5).

Study Year	Ethnicity	Study Design	Diagnostic Criteria	Control	Case/Control (n)	Mean age of Case/Control	Genotyping Method
Gupta et al., 2010	Indian	Case-Control	WHO	Normal glucose tolerance subjects	219/184	57.9/53.8	Sequencing
Chauhan et al., 2010	Indian	Case-Control	WHO	Normal glucose tolerance subjects	1017/1006	48.9/40.1	Golden Gate Assay
Sanghera et al., 2008	Indian	Case-Control	ADA	Normal glucose tolerance subjects	532/386	54.1/51.2	Taq Man
Rees et al., 2011	Pakistani	Case-Control	WHO	Normal glucose tolerance subjects	1663/1567	55.7/55.9	Taq Man
Present study	Indian	Case- Control	WHO	Normal glucose tolerance subjects	400/400	55.2/53.9	TETRA-ARMS

Abbreviations: NS-Not specified, GWAS-Genome Wide Association Study, WHO- World Health Organization, ADA- American Diabetes Association, PCR-SSCP-Polymerase Chain Reaction-Single Strand Conformation Polymorphism, TETRA-ARMS – Amplification Refractory Mutation System, n-Number.

#### Quantitative synthesis

**Figure 2:**
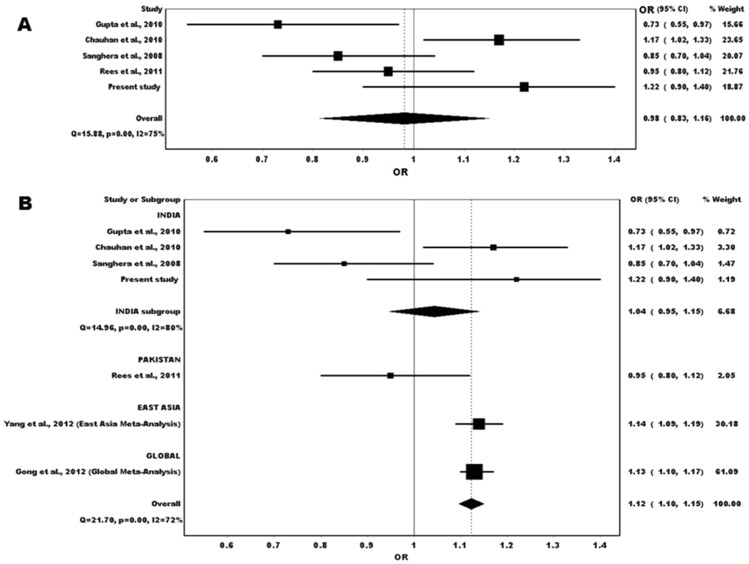
Meta-analysis for the studies of *KCNJ11* polymorphism (rs5219) with T2D. A. Overall pooled OR of South Asian studies. B. Overall pooled OR and ORs within subgroups (South Asian (India and Pakistan) East Asian and Global) were shown. Black squares indicate the individual OR of the studies with size of each square being proportional to the study weighting in the meta-analysis and horizontal line representing the 95% CI. The black colored diamond represents the pooled OR

We observed a high heterogeneity present among the 5 studies with a Cochran's P value of<0.05 and an I^2^ value of 75%. The overall per allele OR of the *KCNJ11* polymorphism (rs5219) under random effects model in South-Asian population showed no association (OR = 0.98, 95%CI = 0.83–1.16). When stratification analysis was done only for the Indian subgroup within the South-Asian population we observed an insignificant risk of *KCNJ11* rs5219 on T2D susceptibility (OR = 1.04, 95% CI = 0.95–1.15). However, when we pooled our data with the other 4 studies we observed a significant association of the *KCNJ11* polymorphism (rs5219) (OR = 1.12, 95% CI = 1.10–1.15) (Figure 2). Our result was consistent with that reported by Yang et al., 2012 (OR = 1.14, 95% CI = 1.09–1.19) and Gong et al., 2012 (OR = 1.13, 95% CI = 1.10–1.17) [30], [17].

#### Sensitivity analysis

When we performed sensitivity analysis to evaluate the weight of each individual study on the pooled OR by sequential removal of individual studies, there was no one study that could significantly affect the pooled OR (Table 8).

**Table 8:** Sensitivity analysis to evaluate the weight of individual study on pooled odds ratio.

Excluded Study	Pooled OR	Cochran Q	Chi-square	I^2^
Gupta et al., 2010	1.03 (0.8–1.2)	10.01	0.018	70.05
Chauhan et al., 2010	0.93 (0.7–1.1)	9.40	0.024	68.08
Sanghera et al., 2008	1.01 (0.8–1.2)	11.95	0.008	74.90
Rees et al., 2011	0.98 (0.7–1.2)	14.95	0.002	79.94
Present study	0.93 (0.7–1.1)	13.00	0.005	76.92

I^2^ value of<25% indicate low heterogeneity, 25–50%moderateheterogeneity, 50–75% high heterogeneity and>75% extreme heterogeneity.

## Discussion

Protein encoded by *KCNJ11* has a decisive function in insulin secretion from pancreatic β cells, thus making it a potential susceptibility gene for T2D. Large studies of susceptibility gene polymorphisms can offer insight into the association between candidate genes and polymorphisms. *KCNJ11* polymorphisms rs5219 (E23K), rs1800467 (L270V), rs5215 (V337I), rs41282930 (S385C) are the most widely studied. Until now, association of these variants with T2D has been fully investigated only in European populations. Previous reports have shown that individuals may be predisposed to the development of T2D if they carry risk alleles which may predispose to impaired insulin secretion and insulin resistance [35]. Initial reports were inconsistent for the association between *KCNJ11* polymorphism (rs5219) and T2D in Caucasian populations [13]–[15] but later studies of Hani et al.,(1998), Gloyn et al., (2003) and Love et al., (2003) which had larger sample sizes, established this association [5], [7], [8]. Florez et al., (2004) and Barroso et al., (2003) confirmed the association along with other variants such as L270V (rs1800467), rs5218, 3p + 215(rs5210) of *KCNJ11* with T2D [12], [15]. Recently, GWAS studies have confirmed the association of *KCNJ11* polymorphism (rs5219) with T2D in Caucasians with an overall risk allele (OR = 1.14, 95% CI = 1.10–1.19)[36]–[38]. However, studies on the association between *KCNJ11* polymorphism (rs5219) and T2D gave contradictory results in Asian populations. Considering functional importance of the gene and since there are a) very few studies explaining the role of *KCNJ11* polymorphisms (rs5219 (E23K), rs1800467 (L270V), rs5215 (V337I), rs41282930 (S385C)) in South Asian population, b) no reported large scale GWAS studies and c) lack of comprehensive meta-analysis, we have performed a case–control association study with 400 T2D cases and 400 controls and a systematic review and meta-analysis to provide an assessment of the risk association between *KCNJ11* polymorphism (rs5219) and T2D in South Asian population. Five studies were included in the current meta-analysis (including our present study) with 3,831 cases and 3,543 controls. Our case-control association study established that among all the SNPs we evaluated, *KCNJ11* polymorphism (rs5215) was only one that was associated with risk of T2D in South Indian population. The important finding of the study is that rs5219, rs41282930, rs1800467 are not independently associated with T2D. However, they showed a statistically significant association with susceptibility to T2D as two-loci (rs5219 and rs1800467) and haplotype (C-G-C-C). According to our knowledge this is the first report in South-Asian populations revealing the possible relation between the two loci (rs5219 and rs1800467) and the susceptibility of T2D. Genome analysis of *KCNJ11* gene from UCSC genome browser (http://genome.ucsc.edu/) suggested presence of CNV within exon 2 encompassing rs5219 SNP. Therefore, we analyzed CNVs in this region which suggested absence of any such change in majority of samples but however two T2D samples in a total of 50 T2D samples and 50 control samples did show copy number loss, relevance of such a change remains to be clearly understood. Our meta-analysis of South Asian population also showed no significant association of *KCNJ11* polymorphism (rs5219) with risk of T2D. The minor allele frequency (MAF) of control subjects in South Asians (0.28) is much lower than that of East Asians (0.37). Thus a considerable difference in MAF between East Asians and South Asians substantiates the compelling contribution of *KCNJ11* polymorphism (rs5219) to T2D. To dissect the consequence of ethnic differences on susceptibility to T2D with respect to this variant we compared the results of our meta-analysis to previous meta-analyses of East Asian and global populations by Yang et al., 2012 and Gong et al., 2012[30], [17] and we also conducted a stratified population analysis. The overall population analysis with South Asians, East Asians and global population combined showed a significant association with T2D but the positive result was not replicated in the South Asian sub-group analysis. The meta-analysis of East Asian population and global population showed an OR = 1.14, 95% CI = 1.09–1.13 and OR = 1.13, 95% CI = 1.10–1.15 respectively which was similar to the combined overall OR = 1.12, 95% =  1.10–1.15. However, there are some limitations in this meta-analysis that should be kept in mind when interpreting the results. Firstly, there is a high level of population heterogeneity among the studies and interpretation of results. To minimize these, published studies were searched using stringent criteria for inclusion, but still consider able inter-study heterogeneity existed in most of the comparisons. This heterogeneity can be due to age distribution of cases and controls, lifestyle habits and sources of control subjects. Secondly, results of our analysis were based on unadjusted estimates and more accurate analysis could be possible if individual raw data would have been available. Thirdly, lack of data prevented us from identifying interactions between genetic variations and metabolic traits. In conclusion our findings suggest that *KCNJ11* rs5219 gene polymorphism is not an independent risk factor for T2D but in combination with rs1800467 exhibits a risk to the development of T2D in South Indians. Our meta-analysis results also showed no association of *KCNJ11* polymorphism (rs5219) with risk of T2D in South Asian population with an increased overall risk in East Asian and global population, the influence of ethnicity on predisposition to T2D is further confirmed. Since the associations what we have observed in the present study are at a statistical level, more of independent case-control association studies are required to validate these findings in South Asian populations. Further gene-gene interactions and gene-environment interactions should be considered to gain deeper understanding of the genetics of T2D in general and role of *KCNJ11* in particular to the onset of T2D.

## Supporting Information

Table S1 List of primer sequences and amplicon size for the SNPs in the study. Abbreviations: FOP- Forward outer primer (5′-3′), ROP-Reverse outer primer (5′-3′), FIP- Forward inner primer, RIP-Reverse inner primer.
(DOCX)

Table S2 Study quality based on the Newcastle-Ottawa scale.
(DOCX)

Checklist S1 (DOCX)
