# Supplementary material for: Population Specific Impact of Genetic Variants in KCNJ11 Gene to Type 2 Diabetes: A Case-Control and Meta-Analysis Study
Source: PLoS One. 2014 Sep 23;9(9):e107021. doi: 10.1371/journal.pone.0107021 (PMC4172481; doi:10.1371/journal.pone.0107021)
Supplement: Table S1 — List of primer sequences and amplicon size for the SNPs in the study. Abbreviations: FOP- Forward outer primer (5′-3′), ROP-Reverse outer primer (5′-3′), FIP- Forward inner primer, RIP-Reverse inner primer. (DOCX) [file pone.0107021.s001.docx]

**Table S1. List of primer sequences and amplicon size for the SNPs in the study**

| Gene, rsID  Polymorphism | Genotyping method | Product size | Primer sequence |
| --- | --- | --- | --- |
| *KCNJ11* rs5219  C/T | TETRA-ARMS | Product size for OP: 349  Product size for C allele:162  Product size for T allele:237 | FOP- ATGAGCCACCAGGCCATGGCGAAGAG  ROP- AGTGAGGCCCTAGGCCACGTCCGAGG  FIP-(C:Allele)- CTGGCGGGCACGGTACCTGGGATC  RIP-(T:Allele)- GACACGCCTGGCAGAGGACCCTGACA |
| *KCNJ11* rs5215  G/A | TETRA-ARMS | Product size for OP: 391  Product size for G allele: 192  Product size for A allele:250 | FOP-GCTACATACCACATGGTCCGTGTGTAC  ROP-TCGAGATCATCGTCATCCTGGAAG  FIP-(G:Allele)- GCAGAGTGGTGTGGGCACTTTTAG  RIP-(A:Allele)-TGGACTACTCCAAGTTTGGCAACAACT |
| *KCNJ11* rs41282930  C/G | TETRA-ARMS | Product size for OP:369  Product size for C allele:191  Product size for G allele:230 | FOP- TAACCCAGTACAGGTTCCTGCTGAGGCC  ROP- CTCCAAGTTTGGCAACACCGTCAAAGTG  FIP-(C:Allele)- CATGGCTCAGGACAGGGAATCTGCAC  RIP-(G:Allele)- AAGGCCAAGCCCAAGTTCAGCATGTC |
| *KCNJ11* rs1800467  C/G | TETRA-ARMS | Product size for OP: 342  Product size for C allele: 212  Product size for G allele:179 | FOP- CAAGAGCATGATCATCAGCGCCACCA  ROP- GCTACAATGGGCACAAAGCGCTGGC  FIP-(C:Allele)- GGTGCAGGTCGCTGGGTGCGAG  RIP-(G:Allele)-TTGATGCCAACAGCCCACTCTACGTCG |

**Abbreviations: FOP- Forward outer primer (5’-3’), ROP-Reverse outer primer (5’-3’), FIP- Forward inner primer, RIP-Reverse inner primer**
