# Supplementary material for: Population Specific Impact of Genetic Variants in KCNJ11 Gene to Type 2 Diabetes: A Case-Control and Meta-Analysis Study
Source: PLoS One. 2014 Sep 23;9(9):e107021. doi: 10.1371/journal.pone.0107021 (PMC4172481; doi:10.1371/journal.pone.0107021)
Supplement: Table S2 — Study quality based on the Newcastle-Ottawa scale. (DOCX) [file pone.0107021.s002.docx]

# Table S2. Study quality based on the Newcastle-Ottawa scale

| Author | Is the case definition adequate? | Representativeness of the Cases | Selection of Controls | Definition of Controls | Comparability of Cases and Controls on the Basis of the Design or Analysis | Ascertainment of exposure | Same method of ascertainment for cases and controls | Non-Response rate | Total score |
| --- | --- | --- | --- | --- | --- | --- | --- | --- | --- |
| Gupta et al.,2010 | ★ | ★ | ★ | ★ | ★☆ | ★ | ★ | ☆ | 7 |
| Chauhan et al., 2010 | ★ | ★ | ★ | ★ | ★☆ | ★ | ★ | ☆ | 7 |
| Sangheraet al., 2008 | ★ | ★ | ★ | ★ | ★☆ | ★ | ★ | ☆ | 7 |
| Reeset al., 2011 | ★ | ★ | ★ | ★ | ☆☆ | ★ | ★ | ☆ | 6 |
